# Detection of Tick-Borne Bacterial and Protozoan Pathogens in Ticks from the Zambia–Angola Border

**DOI:** 10.3390/pathogens11050566

**Published:** 2022-05-10

**Authors:** Yongjin Qiu, Martin Simuunza, Masahiro Kajihara, Joseph Ndebe, Ngonda Saasa, Penjani Kapila, Hayato Furumoto, Alice C. C. Lau, Ryo Nakao, Ayato Takada, Hirofumi Sawa

**Affiliations:** 1 Division of International Research Promotion, International Institute for Zoonosis Control, Hokkaido University, N 20 W 10, Kita-ku, Sapporo 001-0020, Japan; h-sawa@czc.hokudai.ac.jp; 2 Department of Disease Control, School of Veterinary Medicine, The University of Zambia, P.O. Box 32379, Lusaka 10101, Zambia; martin.simuunza@unza.zm (M.S.); j.ndebe@yahoo.com (J.N.); nsaasa@gmail.com (N.S.); penjanikapila@yahoo.com (P.K.); 3 Division of Global Epidemiology, International Institute for Zoonosis Control, Hokkaido University, N 20 W 10, Kita-ku, Sapporo 001-0020, Japan; kajihara@czc.hokudai.ac.jp (M.K.); atakada@czc.hokudai.ac.jp (A.T.); 4 Japan International Cooperation Agency in Zambia, P.O. Box 30027, Lusaka 10101, Zambia; h1.smnahk.1scesc@gmail.com; 5 Laboratory of Wildlife Biology and Medicine, Department of Environmental Veterinary Sciences, Faculty of Veterinary Medicine, Hokkaido University, N 18 W 9, Kita-ku, Sapporo 060-0818, Japan; alicelau.cc@vetmed.hokudai.ac.jp; 6 Laboratory of Parasitology, Department of Disease Control, Faculty of Veterinary Medicine, Hokkaido University, N 18 W 9, Kita-ku, Sapporo 060-0818, Japan; ryo.nakao@vetmed.hokudai.ac.jp; 7 International Collaboration Unit, International Institute for Zoonosis Control, Hokkaido University, N 20 W 10, Kita-ku, Sapporo 001-0020, Japan; 8 One Health Research Center, Hokkaido University, N 20 W 10, Kita-ku, Sapporo 001-0020, Japan; 9 Division of Molecular Pathobiology, International Institute for Zoonosis Control, Hokkaido University, N 20 W 10, Kita-ku, Sapporo 001-0020, Japan

**Keywords:** *Babesia caballi*, *Candidatus* Midichloria mitochondrii, *Ehrlichia*, *Hepatozoon canis*, *Rickettsia*, *Theileria velifera*, Zambia–Angola border

## Abstract

Tick-borne diseases (TBDs), including emerging and re-emerging zoonoses, are of public health importance worldwide; however, TBDs tend to be overlooked, especially in countries with fewer resources, such as Zambia and Angola. Here, we investigated *Rickettsia*, *Anaplasmataceae*, and Apicomplexan pathogens in 59 and 96 adult ticks collected from dogs and cattle, respectively, in Shangombo, a town at the Zambia–Angola border. We detected *Richkettsia africae* and *Rickettsia aeschilimannii* in 15.6% of *Amblyomma variegatum* and 41.7% of *Hyalomma truncatum* ticks, respectively. *Ehrlichia minasensis* was detected in 18.8% of *H. truncatum*, and *Candidatus* Midichloria mitochondrii was determined in *Hyalomma marginatum*. We also detected *Babesia caballi* and *Theileria velifera* in *A. variegatum* ticks with a 4.4% and 6.7% prevalence, respectively. In addition, *Hepatozoon canis* was detected in 6.5% of *Rhipicephalus lunulatus* and 4.3% of *Rhipicephalus sanguineus*. Coinfection of *R. aeshilimannii* and *E. minasensis* were observed in 4.2% of *H. truncatum*. This is the first report of *Ca.* M. mitochondrii and *E. minasensis*, and the second report of *B. caballi*, in the country. *Rickettsia africae* and *R. aeschlimannii* are pathogenic to humans, and *E. minasensis*, *B. caballi*, *T. velifera*, and *H. canis* are pathogenic to animals. Therefore, individuals, clinicians, veterinarians, and pet owners should be aware of the distribution of these pathogens in the area.

## 1. Introduction

Ticks are important blood-sucking arthropods in medical and veterinary science, second to mosquitos. They not only cause anemia in their hosts, but also carry and transmit a broad range of viruses, bacteria, and protozoa. Some of these microorganisms cause tick-borne diseases (TBDs), which include emerging and re-emerging infectious diseases [1,2]. To date, TBDs have been considered a focal point for human and animal health worldwide. The identification of novel viral and bacterial TBD-causing agents has increased in recent times [3]. An example of emerging TBD agents is *Borrelia fainii*, which was first isolated from a febrile patient in Zambia in 2019 [4]. *Ornithodoros faini* ticks and *Rousettus aegyptiacus* bats are considered as a vector and natural reservoir of *Borrelia fainii*, respectively [4]; however, TBDs tend to be overlooked, especially in low-resource countries, because of limitations in diagnostic infrastructure.

Tick-borne bacterial pathogens include *Rickettsia*, *Anaplasma*, *Ehrlichia*, *Coxiella*, *Orientia*, and *Borrelia*. Among them, *Rickettsia* are obligate intracellular Gram-negative bacteria, and are recognized as the causative agents of important emerging TBDs [5,6]. The symptoms of human rickettsiosis include chills, high fever, headache, skin rash, and photophobia [7]. Species of the agents of human rickettsiosis differ region-wise. For example, *R. japonica* causes Japanese spotted fever prevalent in East Asia, *R. parkeri* causes American Boutonneuse Fever in the USA, and *R. africae* causes African tick bite fever in Africa [8,9,10,11]. Furthermore, *Anaplasma* and *Ehrlichia* are obligate intracellular bacteria belonging to the family *Anaplasmataceae*. Some of these bacteria cause TBDs in humans and animals. For example, *A. phagocytophilum* causes human granulocytic anaplasmosis and has been reported worldwide, including in Africa [12,13,14]. *Anaplasma platys* has primarily been isolated from dogs with cyclic thrombocytopenia; it has also been reported in Africa [15]. Importantly, human infection with *A. platys* has also been reported in Venezuela and South Africa [16,17].

The common tick-borne protozoan pathogens are members of the phylum Apicomplexa and belong to the genera *Babesia*, *Theileria*, and *Hepatozoon*. *Babesia microti*, *B. divergens*, *B. venatorum*, and *B. duncani* are the major etiological agents of human babesiosis. Most human cases of babesiosis have been reported in the USA, but this disease has also been reported in Asia, Africa, Australia, Europe, and South America [18]. *Babesia gibsoni*, *B. canis*, *B. rossi*, and *B. vogeli* are widely known as causative agents of canine babesiosis [19]. *Babesia bigemina* and *B. bovis* are agents of bovine babesiosis [20,21]. *Theileria* species, particularly *T. annulata* and *T. parva*, have caused the most significant economic losses in livestock production worldwide. *Theileria annulata* causes tropical theileriosis in several tropical regions in southern Europe, northern Africa, and Asia [22]. Conversely, *T. parva* causes East Coast fever, which is distributed in the eastern, central, and southern parts of Africa [23]. *Hepatozoon canis* and *H. americanum* have been reported to cause canine and feline hepatozoonoses worldwide, which are the most common and important tick-borne hepatozoonoses [24].

Studies on tick-borne pathogens in Zambia, such as *Rickettsia*, *Anaplasmataceae*, and Apicomplexa, have primarily been conducted in the southern, central, and eastern parts of the country [15,25,26,27,28,29,30,31]. Angola is a neighboring country and shares borders with the western region of Zambia. A few studies on tick-borne pathogens have also been reported in Angola, primarily in the central and western regions [31,32]. Geographically, wildlife can easily pass through the Zambia–Angola border, and ticks might be attached to the bodies of animals during transit. Therefore, the investigation of ticks and tick-borne pathogens in the Zambia–Angola border may provide valuable information for a better understanding of the distribution of TBDs in western Zambia and eastern Angola. In this study, we performed the molecular-level screening and characterization of *Rickettsia*, *Anaplasmataceae*, and Apicomplexa detected from ticks in Shangombo at the Zambia–Angola border.

## 2. Results

### 2.1. Identification of Tick Species

**Figure 1:**
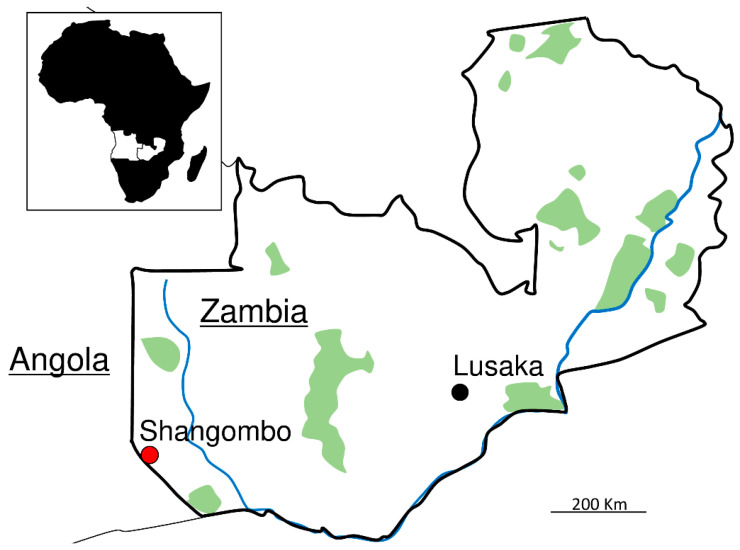
Map of the sampling site. The red and black dots are sampling place and capital city, respectively.

Overall, we collected 59 and 96 adult ticks infesting dogs and cattle, respectively, in Shangombo, a town in the Zambia–Angola border region (Figure 1). Morphological identification revealed that 2 *Amblyomma variegatum* (males), 31 *Rhipicephalus lunulatus* (12 females and 19 males), 23 *R. sanguineus* (10 females and 13 males), and 3 *Rhipicephalus* spp. (males) ticks were collected from dogs, and 1 *A. pomposum* (male), 43 *A. variegatum* (7 females and 36 males), 1 *Hyalomma marginatum* (female), 48 *H. truncatum* (14 females and 34 males), and 3 *R. appendiculatus* (females) ticks were collected from cattle (Table 1).

### 2.2. Detection and Characterization of Rickettsia

**Figure 2:**
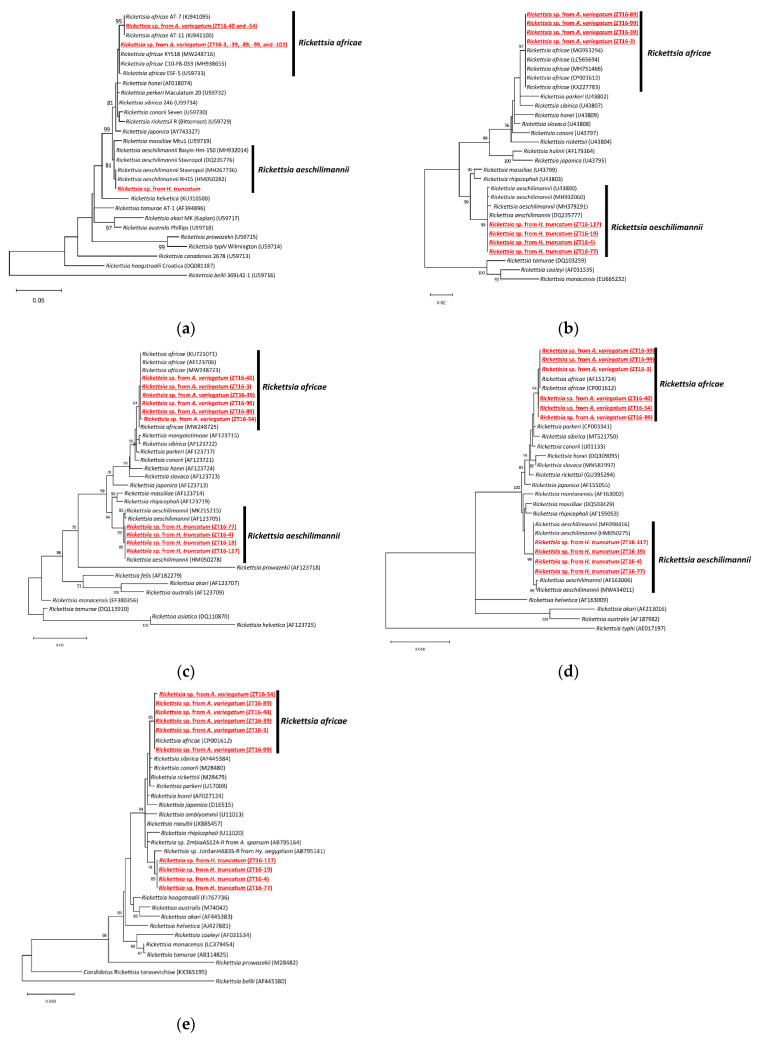
Phylogenetic trees of detected *Rickettsia* spp. based on the sequences of five genes: (**a**) *gltA*; (**b**) *ompA*; (**c**) *ompB*; (**d**) *sca4*; and (**e**) *htrA*. The accession numbers for the nucleotide sequences are provided after the species names. The analyses were performed using the maximum likelihood method. Bootstrap values >70% based on 1000 replications are indicated on the interior branch nodes.

Ticks infesting cattle were used for screening *Rickettsia* spp. using a polymerase chain reaction (PCR) targeting the *gltA* gene. As a result, *Amblyomma variegatum* (*n* = 7) and *Hyalomma truncatum* (*n* = 20) were positive for *Rickettsia* spp., representing three sequence variants. Sequence variants 1 and 2 identified from *A. variegatum* showed 100% identities to *Rickettsia africae* clones AT-11 and C10-F8-303, respectively, while sequence variant 3 identified from *H. truncatum* showed a 100% identity to *Rickettsia aeschlimannii* (Figure 2). Prevalence of *R. africae* in *A. variegatum* and *R. aeschlimannii* in *H. truncatum* were 15.6% and 41.7%, respectively.

### 2.3. Detection and Characterization of Anaplasmataceae

**Figure 3:**
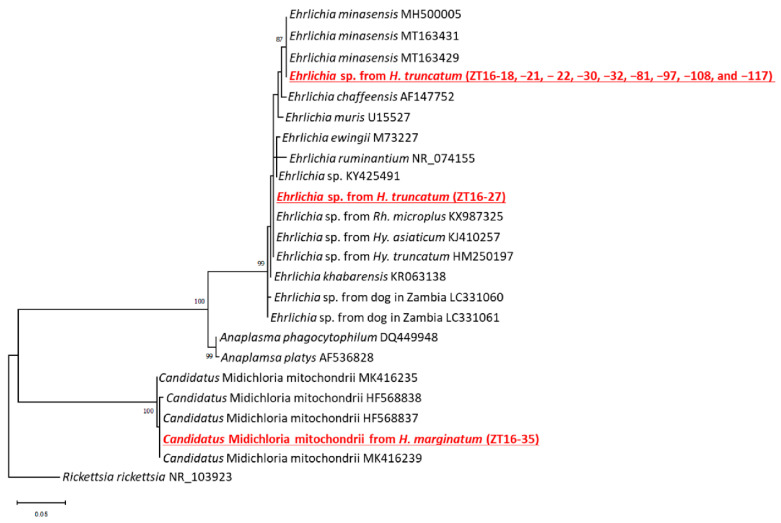
Phylogenetic trees of *Anaplasmataceae* based on partial 16S ribosomal DNA sequences (305 bp). The analysis was performed using the maximum likelihood method. Bootstrap values >70% based on 1000 replications are shown on the interior branch nodes.

For the screening of *Anaplasmatacea*, 59 ticks from dogs and 96 ticks from cattle were used. *Hyalomma truncatum* (*n* = 10) and *H. marginatum* (*n* = 1) were positive for *Anaplasmataceae*, representing three sequence variants. Sequence variants 1 and 2 identified from *H. truncatum* showed 100% identities to *Ehrlichia* sp. and *Ehrlichia minasensis*, respectively, while sequence variant 3 identified from *H. marginatum* showed a 100% identity to *Candidatus* Midichloria mitochondrii (Figure 3). The prevalence of *Ehrlichia* sp. and *E. minasensis* in *H. truncatum* were 2% and 18.8%, respectively, while the prevalence of *Ca.* Midichloria mitochondrii in *H. marginatum* was 100%.

### 2.4. Detection and Characterization of Apicomplexa

**Figure 4:**
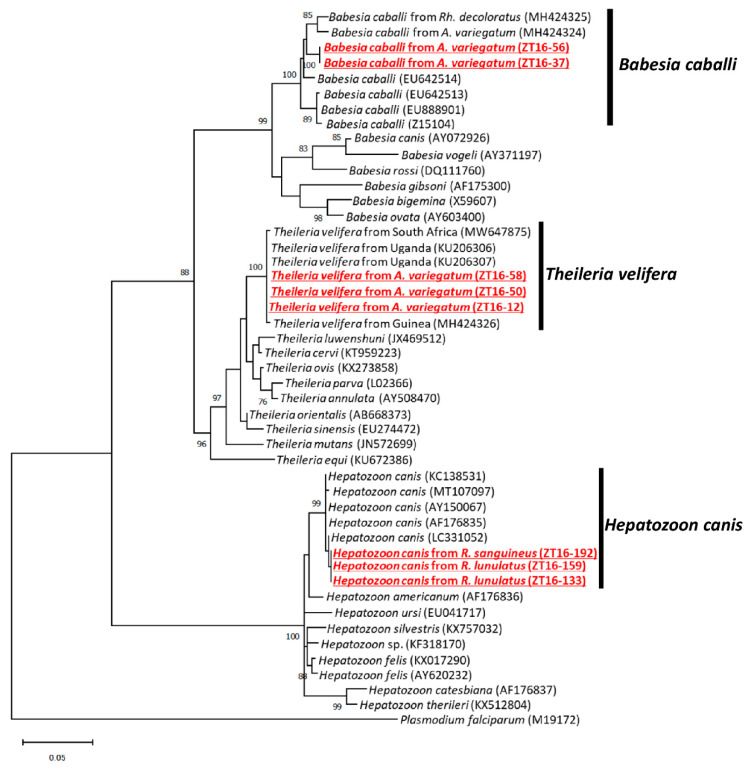
Phylogenetic tree of the detected protozoa based on the partial 18S ribosomal DNA sequences. The accession numbers for the nucleotide sequences are mentioned after the species names. The analyses were performed using the maximum likelihood method. Bootstrap values >70% based on 1000 replications are presented on the interior branch nodes.

The same ticks collected from dogs and cattle were used to screen Apicomplexa. As a result, *Rhipicephalus lunulatus* (*n* = 2), *R. sanguineus* (*n* = 1), and *Amblyomma variegatum* (*n* = 5) were positive for Apicomplexa, representing three sequence variants. Sequence variant 1 identified from *R. lunulatus* and *R. sanguineus* showed a 100% identity to *Hepatozoon canis*. Sequence variant 2 identified from three *A. variegatum* showed a 100% identity to *Theileria velifera*, while sequence variant 3 identified from two *A. variegatum* showed a 98.1% identity to *Babesia caballi* (Figure 4). The prevalence of *H. canis* in *R. lunulatus* and *R. sanguineus* was 6.5% and 4.3%, respectively, while the prevalence of *T. verifera* and *B. caballi* in *A. variegatum* was 6.7% and 4.4%, respectively.

### 2.5. Coinfection

Coinfections of *Rickettsia aeschlimannii* and *Ehrlichia minasensis* were observed from two *Hyalomma truncatum* ticks. None of the tick samples were coinfected with Apicomplexa and *Rickettsia* or *Anaplasmataceae*.

## 3. Discussion

We investigated the presence of *Rickettsia*, *Anaplasmataceae*, and Apicomplexa species in ticks collected from cattle and dogs in Shangombo, a town located at the border of Zambia and Angola. We identified *R. africae*, *R. aeschlimannii*, *E. minasensis*, *Ehrlichia* sp., *Ca.* M. mitochondrii, *H. canis*, *T. velifera*, and *B. caballi*. To the best of our knowledge, this is the first study to report *Ca.* M. mitochondrii and *E. minasensis*, and the second study to report *B. caballi*, in the country.

*Rickettsia africae* detected from *A. variegatum* in this study is widely known as a causative agent of African tick bite fever, which is one of the zoonotic tick-borne fevers from the spotted fever group of rickettsiae of emerging global health concern [33]. In addition, we also detected *Rickettsia aeschlimannii* from *H. truncatum*, which is a human pathogenic rickettsia [34]. Previous epidemiological studies on rickettsia in Zambia were conducted in the central, eastern, and southern parts of the country [25,26,35,36,37,38,39]. Thus, this study is the first evidence of pathogenic rickettsiae in the western part of the country.

*Ehrlichia minasensis* was first isolated from cattle in midwestern Brazil in 2014, and it was experimentally confirmed to be an agent of clinical ehrlichiosis in calves [40]. To date, *E. minasensis* has been reported worldwide, including in South Africa, Kenya, and Ethiopia [41,42,43]. The primary vectors of *E. minasensis* are *Rhipicephalus microplus* and other *Rhipicephalus* ticks, but it has also been detected in *Amblyomma*, *Hyalomma*, and *Haemaphysalis* ticks [44,45,46,47]. In this study, *E. minasensis* was detected in nine *H. truncatum* ticks for the first time in Zambia. Our results expanded the distribution records of *E. minasensis*, suggesting the likelihood of bovine ehrlichiosis caused by *E. minasensis* occurring in Zambia. Further investigations of *E. minasensis* are warranted to evaluate the current situation in the country.

*Candidatus* Midichloria mitochondrii is an endosymbiont of ixodid ticks, such as *Ixodes ricinus*, *A. americanum*, *H. marginatum*, *R. turanicus*, and *H. wellingtoni*, and has been reported worldwide [48,49,50,51,52]. Recently, it was also reported in the argasid tick, *Ornithodoros turicata* [53]. The role of *Ca.* M. mitochondrii in the host tick is speculated to enhance the host fitness and/or for ensuring its presence in the host population [54]. In this study, we provided the first evidence of *Ca.* M. mitochondrii in *H. marginatum* ticks in Zambia.

We detected *Theileri velifera* and *Babesia caballi* in *A. variegatum*. *Theileria velifera* has been associated with low pathogenic or asymptomatic animal infections in cattle in Africa. Previous studies have reported the detection of *T. velifera* in impalas, buffalos, and cattle in Zambia, and it has been found to show a high prevalence in cattle [55,56], while, *B. caballi* is a pathogenic protozoan found in horses, donkeys, and zebras. Interestingly, *B. caballi* was detected in *A. variegatum* ticks infesting cattle in the Republic of Guinea [57] and was detected in 5.3% (16/299) of cattle blood samples by a reverse line blot hybridization assay in Zambia [55], even though *B. caballi* is known as an equine babesia. Thus, we speculated that a genotype of *B. caballi* is able to infect cattle and be carried by *A. variegatum*; however, further studies on the *B. caballi* in cattle in Zambia are required to evaluate this hypothesis.

*Hepatozoon canis*, an agent of canine hepatozoonosis [24,58], was detected in two *R. lunulatus* and one *R. sanguineus* ticks in the present study. In addition, a previous study in the same area showed a relatively high prevalence of *H. canis* in dogs [15]. Therefore, Shangombo might be an endemic area of *H. canis*. For better vigilance, veterinarians and dog owners residing in an around Shangombo should be aware of the symptoms of canine hepatozoonosis.

*Amblyomma variegatum* is a three-host tick that utilizes different hosts during each life stage. The larva and nymph ticks are generally present in great numbers on small mammals and birds, such as the mongoose and cattle egret. While adult ticks utilize larger mammals, such as camels and cattle. Evidence of cattle egret playing a role in transporting the larvae and nymphs of the tick, and that the dispersal of *A. variegatum* is associated with the migration patterns of the bird have been reported [59,60]. Given this, as well as the detection of *R. africae*, *T. velifera*, and *B. caballi* in *A. variegatum*, these pathogens might be crossing the Zambia–Angola border.

In this study, ticks were collected from dogs and cattle. Therefore, we cannot eliminate the possibility for detecting pathogens in blood meal in ticks, which is the limitation of this study. Further study in ticks collected from pasture in the study area is required to determine the vector ticks of the detected pathogens.

In conclusion, we studied tick-borne bacterial and protozoan pathogens in Shangombo, as there is relatively limited information on tick-borne pathogens in this area. This study provided information on the presence of *R. africae*, *R. aeschlimannii*, *E. minasensis*, *Ca.* M. mitochondrii, *H. canis*, *T. velifera*, and *B. caballi* in the study region. The information may be helpful to researchers and individuals not only from Zambia but also from Angola for preventing TBDs. Further investigation of tick-borne pathogens in the area is necessary to evaluate the prevalence of TBDs in the area.

## 4. Materials and Methods

Ticks were removed using a tick twister (H3D, Lavancia, France) or forceps from dogs and cattle in Shangombo (16.32 S, 22.10 E) (Figure 1), Western province, Zambia, in January 2016. The tick species were identified based on morphological taxonomic keys using a stereomicroscope [61]. The total DNA was extracted from individual ticks using a TRIzol reagent (Invitrogen, Waltham, MA, USA) according to the manufacturer’s instructions.

For screening the rickettsial infections, DNA samples from tick-infested cattle were initially tested using *gltA*-PCR, as previously described [62]. The *gltA*-PCR was performed with the primers gltA_Fc and gltA_Rc, and the 20-μL reaction mixture contained 0.1 μL Ex Taq Hot Start version (Takara Bio Inc., Shiga, Japan), 2 μL 10 × Ex Taq buffer, 1.6 μL 2.5 mM dNTP mixture, 200 nM of each primer, and 2 μL template DNA. UltraPure™ distilled water (Invitrogen) was added as a negative control instead of template DNA. The PCR products were electrophoresed in a 1.2% agarose gel stained with Gel-Red™ (Biotium, Hayward, CA, USA), and visualized with a UV trans-illuminator. When the *gltA*-PCR yielded a positive result, the selected samples were used for further characterization based on the sequences of four additional genes: *ompA*, *ompB*, *sca4*, and *htrA*. The primers used in this study are listed in Table 2.

For the detection and characterization of *Anaplasmataceae*, PCR targeting the 16S rDNA of family *Anaplasmataceae* was performed using the primers EHR16SD and EHR16SR [63]. The universal primer set BTH-1F and BTH-1R, targeting the 18S rRNA gene of *Babesia*–*Theileria*–*Hepatozoon*, was used for the detection and characterization of tick-borne apicomplexans [64].

**Table 2:** Primers used in this study.

Organisms	Gene	Primer Name	Expected Size (bp)	Sequence (5′-3′)	Reference
*Rickettsia*	*gltA*	gltA_Fc gltA_Rc	580	CGAACTTACCGCTATTAGAATG CTTTAAGAGCGATAGCTTCAAG	[62]
	*ompA*	Rr.190.70p Rr.190.602n	530	ATGGCGAATATTTCTCCAAAA AGTGCAGCATTCGCTCCCCCT	[65]
	*ompB*	120_3599 120_2788	816	TACTTCCGGTTACAGCAAAGT AAACAATAATCAAGGTACTGT	[66]
	*sca4*	D1f D928r	928	ATGAGTAAAGACGGTAACCT AAGCTATTGCGTCATCTCCG	[67]
	*htrA*	17K_3 17K_5	552	TGTCTATCAATTCACAACTTGCC GCTTTACAAAATTCTAAAAACCATATA	[68]
*Anaplasmataceae*	16S rDNA	EHR16SD EHR16SR	345	GGTACCYACAGAAGAAGTCC TAGCACTCATCGTTTACAGC	[63]
*Babesia*-*Theileria*-*Hepatozoon*	18S rDNA	BTH-1F BTH-1R	690	CCTGMGARACGGCTACCACATCT TTGCGACCATACTCCCCCCA	[64]

The PCR products were purified using ethanol precipitation or were cloned using the pGEM-T Easy Vector system (Promega, Southampton, Hampshire, UK) and DH5 alpha competent cells (TOYOBO, Osaka, Japan). Cycle sequencing for all amplicons was conducted using the BigDye Terminator version 3.1 chemistry (Applied Biosystems, Foster City, CA, USA). Sequencing products were run on a 3130xl Genetic Analyzer (Applied Biosystems). The DDBJ/EMBL/GenBank accession numbers obtained were LC683090 to LC683109 (See Supplementary Table S1).

Sanger sequencing data from amplified PCR products were analyzed using GENETYX version 9.1 (GENETYX Corporation, Tokyo, Japan). Phylogenetic analysis was conducted using MEGA version X [69]. The sequences were aligned with closely related sequences deposited in the databases (DDBJ/EMBL/GenBank) using ClustalW, and a maximum likelihood phylogram was applied to generate the phylogenetic trees.

## Figures and Tables

**Table 1:** Number of samples used in the study.

Host Species	Tick Species	Female	Male
Dogs	*Amblyomma variegatum*	0	2
	*Rhipicephalus lunulatus*	12	19
	*R. sanguineus*	10	13
	*Rhipicephalus* spp.	0	3
Cattle	*A. pomposum*	0	1
	*A. variegatum*	7	36
	*Hyalomma marginatum*	1	0
	*H. truncatum*	14	34
	*R. appendiculatus*	3	0

## Data Availability

All relevant data are provided in the manuscript.

## Supplementary Materials

The following are available online at https://www.mdpi.com/article/10.3390/pathogens11050566/s1, Table S1: Accession numbers obtained in this study.
